# Commentary: “Nitric oxide releases Cl^−^ from acidic organelles in retinal amacrine cells”

**DOI:** 10.3389/fncel.2015.00401

**Published:** 2015-10-06

**Authors:** Werner Kilb

**Affiliations:** Institute of Physiology, University Medical Center of the Johannes Gutenberg UniversityMainz, Germany

**Keywords:** GABA, nitric oxide, nitrite, amacrine cell, short term plasticity, chloride channels

In their recent article (Krishnan and Gleason, 2015) Vijai Krishnan and Evanna Gleason investigate the cellular mechanisms underlying the shift in the GABA reversal potential upon application of nitric oxide (NO). Functional alteration in GABAergic signaling by alterations in the GABA reversal potential has been identified as an important mechanism of plasticity (Raimondo et al., 2012) and NO is clearly one key substance involved in plasticity (Prast and Philippu, 2001). Therefore, the investigation of the mechanisms behind the NO induced shift in GABAergic effects is an important issue. However, in my opinion the authors neglected a possible explanation of their observations in the discussion section of their recent article.

The detailed investigation in the present article was based on the seminal observation by the same group that moderate concentrations of NO donors induced a slight increase in GABA_A_ receptor mediated responses without affecting the reversal potential, while the application of a saturated NO solution (resulting in “hundreds of nanomolar to low micromolar” NO concentrations) led to a significant shift in the GABA reversal potential (Hoffpauir et al., 2006). The latter effect was independent of the soluble guanylate cyclase, which is elementary for the classical NO signaling pathway, and persisted in nominally Cl^−^-free extracellular solution, suggesting that NO may trigger a Cl^−^ release from intracellular stores (Hoffpauir et al., 2006). Further experiments by the authors suggest that a NO-induced intracellular acidification triggers the release of Cl^−^ from intracellular compartments (McMains and Gleason, 2011). In the present study they used nominally Cl^−^-free extracellular and intracellular (pipette-) solutions to “wash out both cytosolic and protein-bound Cl^−^” and thereby isolate Cl^−^ stores “contained within an intracellular membrane-bound compartment” (Krishnan and Gleason, 2015). These experiments demonstrated that application of NO or NO-donors re-established GABAergic currents under nominally Cl^−^-free conditions, and that this effect was attenuated in the presence of the V-type ATPase inhibitor bafilomycin and was abolished by the uncoupling agent FCCP. From these experimental findings they came to the well supported conclusion, that acidic compartments are a possible source for the NO-induced increase in intracellular Cl^−^ anions.

However, when such high NO concentrations are used, it should be considered that the mitochondrial cytochrome c oxidase can reduce NO to nitrite (NO${}_{2}^{-}$), although no information about the maximal rates for this process is available (Sarti et al., 2012). The permeability of NO${}_{2}^{-}$ through GABA_A_ receptors is higher than their Cl^−^ permeability (Bormann et al., 1987). Thus the intracellular generation of NO${}_{2}^{-}$ might contribute to the re-occurrence of inward currents after NO application under nominally Cl^−^-free solutions. Notably, the oxidation of NO to NO${}_{2}^{-}$ relies on functional oxidative phosphorylation (Sarti et al., 2012). Thus, the reduction of NO-induced GABA currents by bafilomycin and its abolishment by FCCP (Krishnan and Gleason, 2015) may be caused by the fact, that both substances induce mitochondrial dysfunction via uncoupling (Zhdanov et al., 2012).

Therefore, I suggest that the authors should consider the formation of NO${}_{2}^{-}$ as an additional hypothesis to explain their observations. To differentiate between both hypotheses, a conclusive experiment would be to test whether the NO-induced GABA currents in nominally Cl^−^-free intra- and extra-cellular solutions are depletable (indicating a limited intracellular reservoir of Cl^−^ ions) or whether repetitive NO applications induced sustained GABA currents (indicating a NO-dependent generation of anions permeable via the GABA_A_ receptor).

## Funding

This work was funded by DFG grant KI835/2 to WK.

### Conflict of interest statement

The author declares that the research was conducted in the absence of any commercial or financial relationships that could be construed as a potential conflict of interest.
